# The role of neuropeptide somatostatin in the brain and its application in treating neurological disorders

**DOI:** 10.1038/s12276-021-00580-4

**Published:** 2021-03-19

**Authors:** You-Hyang Song, Jiwon Yoon, Seung-Hee Lee

**Affiliations:** Department of Biological Sciences, Korea Advanced Institute of Science and Technology, 291 Daehak-ro, Yuseong-gu, Daejeon 34141 Republic of Korea

**Keywords:** Molecular neuroscience, Drug development

## Abstract

Somatostatin (SST) is a well-known neuropeptide that is expressed throughout the brain. In the cortex, SST is expressed in a subset of GABAergic neurons and is known as a protein marker of inhibitory interneurons. Recent studies have identified the key functions of SST in modulating cortical circuits in the brain and cognitive function. Furthermore, reduced expression of SST is a hallmark of various neurological disorders, including Alzheimer’s disease and depression. In this review, we summarize the current knowledge on SST expression and function in the brain. In particular, we describe the physiological roles of SST-positive interneurons in the cortex. We further describe the causal relationship between pathophysiological changes in SST function and various neurological disorders, such as Alzheimer’s disease. Finally, we discuss potential treatments and possibility of novel drug developments for neurological disorders based on the current knowledge on the function of SST and SST analogs in the brain derived from experimental and clinical studies.

## Neuropeptide: Nerve cell protein may provide treatment options

Developing stable analogues of a key neuronal protein and finding ways to deliver them directly to the brain may provide novel treatments for neurological disorders. The neuropeptide somatostatin (SST) is involved in regulating circuits in the brain cortex and maintaining cognitive function. Reduced SST expression is a recognised feature of brain disorders including Alzheimer’s disease. Seung-Hee Lee and co-workers at the Korea Advanced Institute of Science and Technology, Daejeon, South Korea, reviewed the role of SST and examined its therapeutic potential. SST deficiency appears to cause the significant memory loss found in Alzheimer’s, and there has been some success trialling intravenous injection of SST in patient trials. However, SST is short-lived in the body, limiting its usefulness as a treatment. Stable SST analogues and safe delivery methods could broaden treatment options for multiple brain conditions.

## Introduction

In the mammalian cerebral cortex, excitatory and inhibitory neurons (INs) are intermingled and balance network activity to allow processing of cognitive information. INs, which constitute 20–30% of neurons in the cortex, exert local GABAergic inhibition to regulate the firing activity of cortical neurons^1^ and balance network activity^2^. The precise morphological, electrophysiological, and neurochemical characteristics of INs have been determined, and distinct types of INs have been identified^3^. Specifically, INs can be classified into three nonoverlapping subgroups on the basis of neurochemical properties: parvalbumin- (PV^+^), somatostatin- (SST^+^), and 5-HT_3A_ receptor-expressing (5HT_3A_R^+^) INs^4^. PV^+^ and SST^+^ INs are the two major subgroups of INs in the cortex, comprising ~80% of all GABAergic interneurons. Interestingly, PV^+^ INs are known to exert perisomatic inhibition, while SST^+^ INs exert dendritic inhibition. Dendritic computation of synaptic inputs is crucial for integration of thousands of synaptic inputs from other neurons^5^. Thus, dendritic inhibition by SST^+^ INs in the cortex is particularly interesting. Previous studies have revealed that SST^+^ INs receive long-range inputs^6^, exert local inhibition to balance excitatory synaptic inputs, and modulate cortical processing^7^. Furthermore, SST^+^ INs express the neuropeptide SST along with GABA, and these two neurotransmitters can be coreleased upon activation.

In addition to these neurochemical differences, SST^+^ INs show unique electrophysiological properties compared to other types of neurons. On average, the spike width of SST^+^ INs is broader than that of PV^+^ INs but narrower than that of excitatory neurons^8^. Moreover, while PV^+^ INs show fast-spiking activity and pyramidal neurons (PNs) show regular-spiking activity, there are heterogeneous populations of SST^+^ INs that exhibit diverse discharge patterns. They show distinct spiking responses according to the cortical layer in which they reside. The majority of SST^+^ INs located in the infragranular cortical layers show low-threshold spiking (LTS) because of the long calcium-mediated plateau^4^. LTS cells show after hyperpolarizations, exhibiting a triphasic waveform composed of early and late peaks. On the other hand, supragranular SST^+^ INs show regular-spiking activity with frequency adaptation. These cells are referred to as regular-spiking nonpyramidal (RSNP) cells^9,10^. In addition to the subtypes of SST^+^ INs mentioned above (LTS and RSNP cells), smaller but diverse subsets of SST^+^ INs that exhibit nonaccommodating firing patterns analogous to the fast-spiking activity of PV^+^ INs^11^, burst irregular spiking, or stuttering responses also exist^10,11^. The diverse physiological properties of SST^+^ IN subtypes supposedly indicate their various functions in the cortex.

However, it is still unclear whether all subtypes of SST^+^ INs play different roles in modulating cortical processing by releasing the neuropeptide SST. The following questions remain: which subtypes of SST^+^ INs release SST (a subset of or the entire population of SST^+^ INs) and when is SST released in cortical circuits. Although future studies are needed to answer these questions, we summarized the current knowledge on the specific function of the neuropeptide SST in modulating cortical processing in this review. This knowledge can be linked to the understanding of the physiological role of SST^+^ INs in the cortex and may be applied to develop drugs that can mimic the function of SST.

## Functional roles of SST^+^ INs in the cortex

SST^+^ INs are involved in cortical processing in multiple ways. First, SST^+^ INs modulate excitatory inputs to the sensory cortex during sensory processing. When bottom-up sensory inputs arrive in layer 4 of the sensory cortex via the thalamus, the higher-order cortex gives feedback to distal dendrites of neurons in the superficial layers. Bottom-up (from the thalamus) and top-down (from the higher cortex) inputs to the sensory cortex can be modulated by neighboring SST^+^ INs, as SST^+^ INs predominantly innervate the distal dendrites of PNs and exert feedback or feed-forward inhibition of PN activity^12^. Such inhibition by SST^+^ INs results in surround suppression of sensory cortical neurons^13^, top-down modulation of visual processing in the primary visual cortex (VISp) through increased visual gain^14^, and shaping of auditory processing in the auditory cortex^15^. In addition, SST^+^ INs in layer 4 contribute to the disinhibition of PNs by inhibiting PV^+^ INs that exert perisomatic inhibition of PNs in the sensory cortex^16^. Therefore, SST^+^ INs do not simply suppress sensory information but gate the flow of information through PNs. SST^+^ INs also play a pivotal role in the generation of cortical slow waves^17^, which is the hallmark of nonrapid eye movement (NREM) sleep. Upon the activation of the SST^+^ INs, there appears to be an increase in slow-wave activity and NREM sleep duration. This finding suggests the existence of a causal link between the activity of SST^+^ INs and the generation of slow waves during NREM sleep.

Moreover, SST^+^ INs are known to be involved in synaptic plasticity. Their activity can be modulated by the repetition of sensory stimuli or by learning. When animals were continuously exposed to a specific stimulus, the neuronal activity of SST^+^ INs increased, while that of excitatory neurons in layer 2/3 decreased^18^. Conversely, inhibiting SST^+^ INs led to an increase in the activity of layer 2/3 PNs, even after habituation to a specific stimulus. Furthermore, SST^+^ INs play important roles in maintaining temporally the sequential activity of PNs in layer 2/3 of the mouse primary motor cortex during motor training. Activation of SST^+^ INs disrupted the learning-induced temporal shift in sequential activity of PNs and behavioral improvement^19^. Therefore, SST^+^ INs undergo synaptic plasticity during learning and contribute to maintaining the neural activity that represents learned information. Finally, SST^+^ INs are also known to play an important role in maintaining spatial working memory by selective modulation of nearby PNs^20^. Optogenetic activation of dmPFC SST^+^ INs impaired the behavioral performance of mice in memory-guided tasks^21^.

## Functional roles of the neuropeptide SST and its receptors

**Fig. 1:**
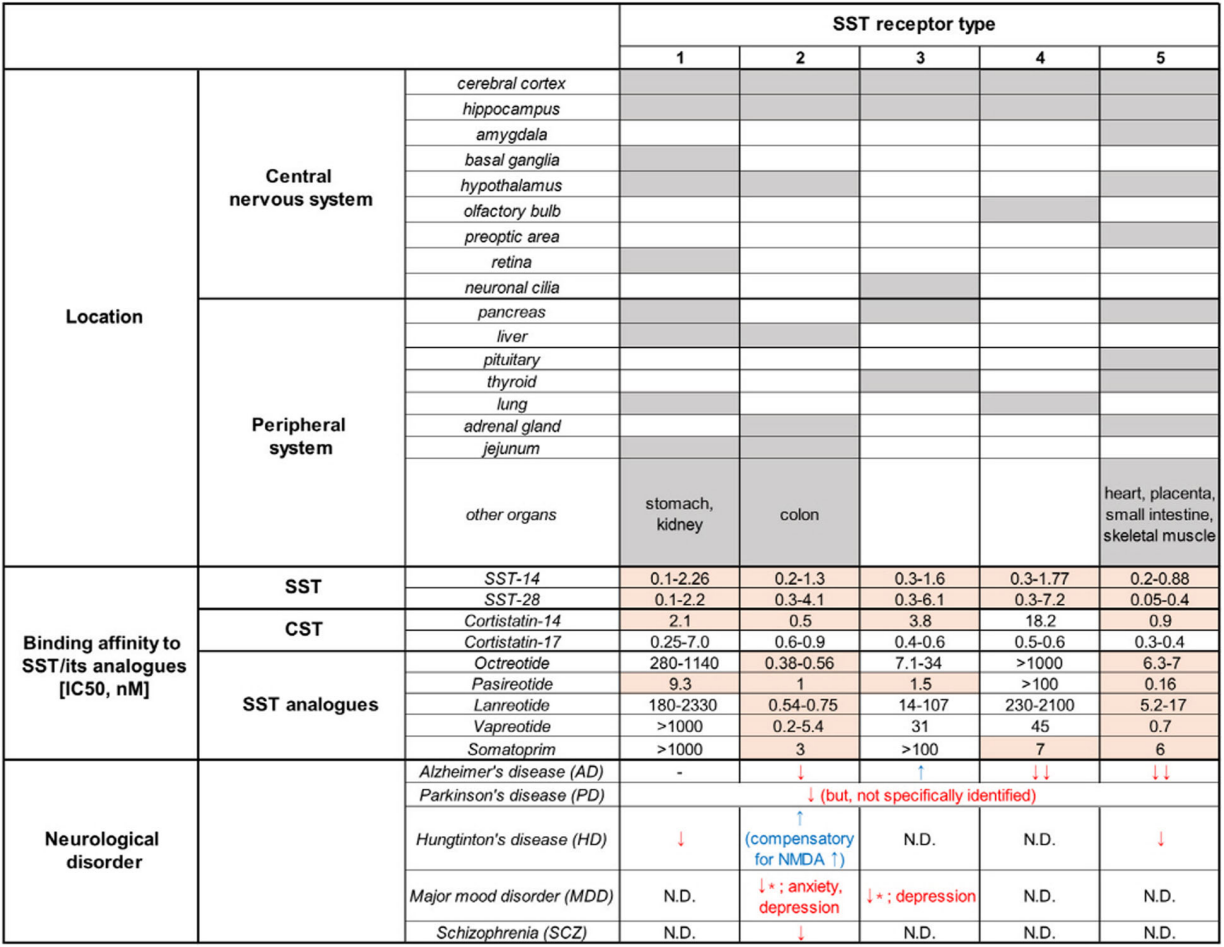
Overview of the properties of SSTR family members: expression localization, binding affinity for SST and its analogs, and associations with neurological disorders. Various subtypes of SSTRs are distributed differently across the central nervous system and the peripheral nervous system^23^. Gray shades indicate the existence of the receptor subtypes in the corresponding area. Red shades indicate receptor subtypes with a strong binding affinity (IC_50_ < 10 nM). Note that SST shows a high binding affinity for all SSTR subtypes. CST resembles SST and has a similar strong binding affinity for all SSTR subtypes. SST analogs have selective binding properties for certain subtypes of SSTRs and sometimes have higher affinity for SSTRs than SST. Alterations in SSTR expression levels are observed in the neurological disorders discussed in this paper. It is well known that the expression levels of SSTRs are altered in AD patients^42^, whereas the SSTR expression level in other disorders is less clear.

The neuropeptide SST was first isolated from sheep hypothalamic extracts in 1973 and named SRIH (somatotropin-release inhibiting hormone). There are two types of biologically active SST isoforms that vary in constituent amino acids: SST-14 and SST-28. Generally, SST-14 is more predominant in the central nervous system (CNS), whereas SST-28 is more abundant in peripheral body organs^22^. SSTs have been found not only in the nervous system but also in various organs, such as the pancreas, gut, and immune cells. When secreted from these organs, SSTs travel throughout the body via the circulatory system. There are a total of five SST receptor (SSTR) subtypes (SSTR1–5), which are expressed in various parts of the body with distinct distribution patterns (Fig. 1). All SSTRs are G-protein-coupled receptors (GPCRs) with seven transmembrane domains and have nanomolar affinity for both SST-14 and SST-28 (ref. ^23^).

While SSTR5 has a higher binding affinity for SST-28 than SST-14, other SSTRs show a weaker affinity for SST-28 than SST-14 (ref. ^23^) (Fig. 1). Binding of SST to SSTRs suppresses the activity of target cells via activation of a G-protein signaling pathway that inhibits exocytosis by reducing the enzymatic activity of adenylate cyclase and the production of cAMPs. One well-known example is the inhibition of the release of pituitary growth hormone (somatotropin) via the activation of SSTRs (SSTR2 and SSTR5) by SST. In addition, induction of insulin secretion (SSTR5), proliferation inhibition (SSTR3), glucagon secretion (SSTR2), and immune responses (SSTR2) can be achieved by the selective binding of SST to particular SSTRs^24,25^.

**Fig. 2:**
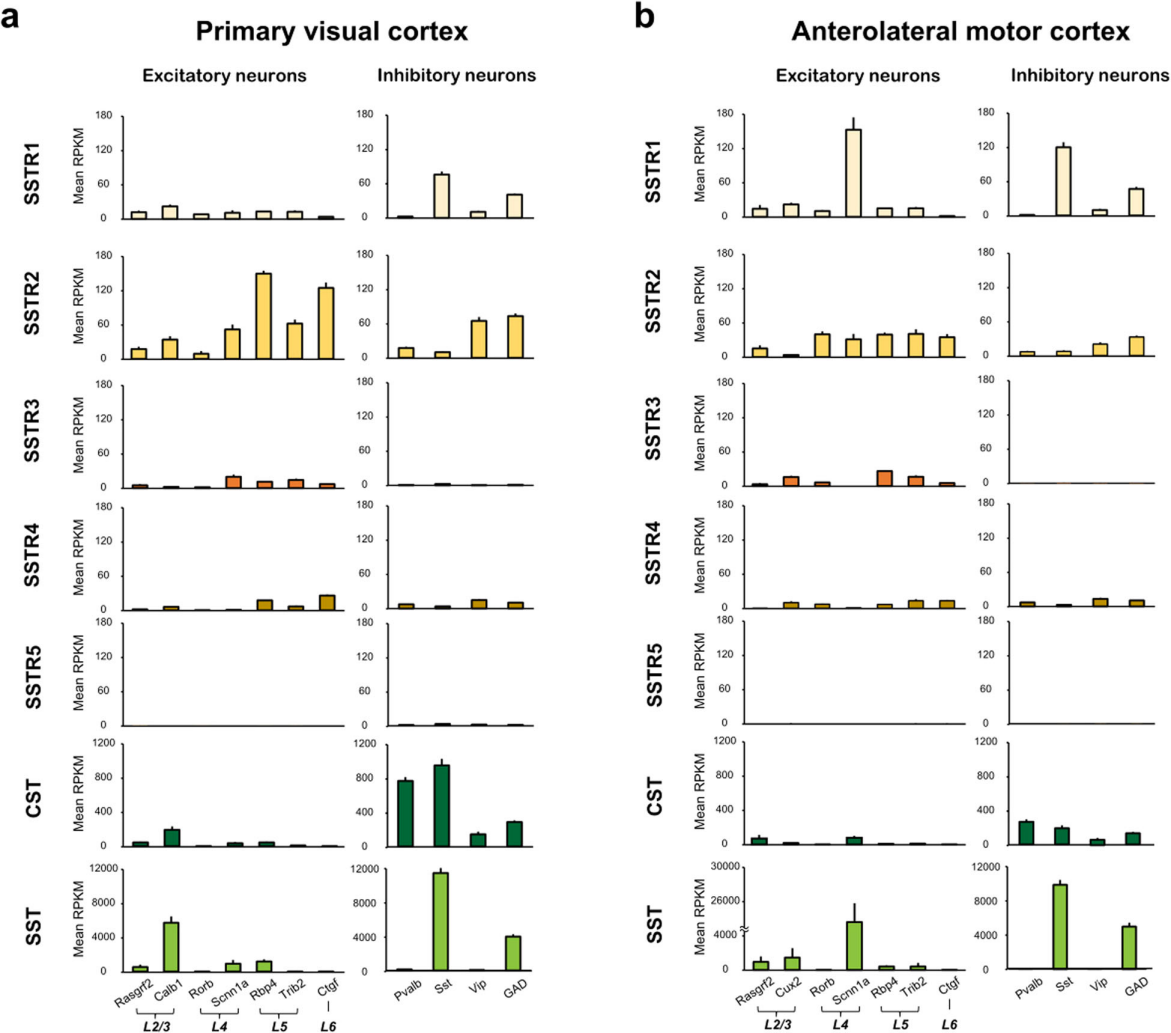
The expression of five types of SSTRs and SST family genes in excitatory and inhibitory neurons in the VISp and ALM. **a** Mean gene expression (RPKM: reads per kilobase of transcript per million mapped reads) of SSTR1–5, CST, and SST in excitatory neurons (left column) and inhibitory neurons (right column) in the VISp. The data are from mouse single-cell RNA sequencing data from the Allen Brain Atlas (total 15,413 cells; version 2018)^28^. The bars represent the mean ± SEM. Genetic markers of the cortical layers were selected based on previous literature^99^. Rasgrf2 Ras protein-specific guanine nucleotide releasing factor 2, Calb1 calbindin1, Rorb RAR-related orphan receptor B, Scnn1α sodium channel epithelial 1 subunit alpha, Rbp4 retinol-binding protein 4, Trib2 Tribbles pseudokinase 2, Ctgf connective tissue growth factor, Pvalb parvalbumin, Sst somatostatin, Vip vasoactive intestinal peptide, GAD glutamate decarboxylase. **b** Same as **a**, but for the ALM (total 10,068 cells). Cux2 was used as a genetic marker of layer 2/3 excitatory neurons instead of Calb1. Cux2 Cut-like homeobox 2.

Several studies have also contributed to revealing the role of the SST and the SSTRs in cortical processing. It has been shown that in rodents, SSTR blockers can impair perceptual task performance. In mice, administration of an SSTR2 agonist to the main olfactory bulb enhanced olfactory discrimination^26^. In addition, a recent study showed that SST enhanced visual processing in the VISp^27^. This study demonstrated that SST reduced excitatory inputs to the PV^+^ INs and improved visual gain in regular-spiking PNs. Serial block-face scanning electron microscopy (SBEM) data confirmed the presence of microcircuits that can mediate the SST-induced suppression of excitatory synaptic transmission to the proximal dendrites of PV^+^ INs^27^. Collectively, SST peptides released from SST^+^ neurons play a critical role in modulating cortical processing of task-relevant sensory information. In a previous study, researchers further examined the expression of various SSTR subtypes in different cell types in the VISp using single-cell RNA sequencing data shared by the Allen Brain Institute^28^. They quantified the mRNA level of SSTR expressed in VISp and anterolateral motor cortex (ALM) neurons (Fig. 2). Consistent with a previous report^27^, SSTR1 was mainly expressed in SST^+^ INs, suggesting that SSTR1 may function as an autoreceptor that can suppress SST^+^ INs. Interestingly, SSTR2 was found to be the most abundantly expressed subtype in the cortex. It was shown to be highly expressed in deep layer excitatory neurons and INs originating from the caudal ganglionic eminence^29^. The same patterns were observed in the VISp and ALM, and these data suggest that the expression patterns of SSTRs are similar across the cortex.

## Release of SST from the neurons

**Fig. 3:**
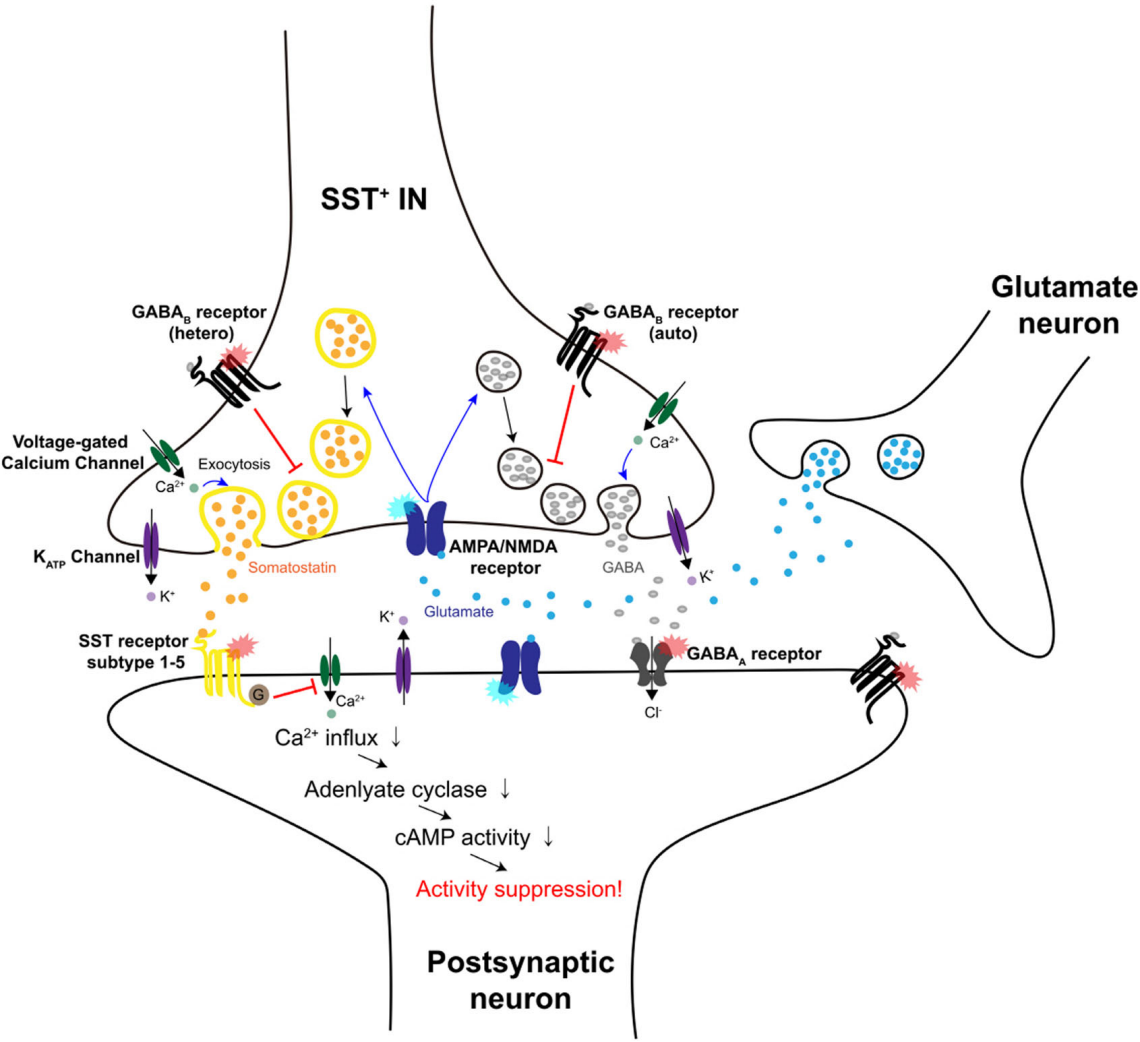
Schematic illustration of SST release in the presynaptic terminal of an SST^+^ IN. In SST-expressing neurons, SST and GABA are known to be colocalized and coreleased. SST (yellow dots) and GABA (gray dots)-containing vesicles are first delivered to the presynaptic terminal and released in a calcium-dependent manner through exocytosis. It has been reported that more time and higher calcium levels are needed to release SST from SST^+^ neurons with higher activity than GABA, as SST is usually delivered in dense-core vesicles (yellow circles). Furthermore, glutamate (blue dots) released from nearby glutamatergic neurons can evoke the release of SST and GABA by activating AMPA/NMDA receptors on axon terminals. Released GABA can inhibit GABA release and SST release through activation of GABA_B_ autoreceptors and heteroreceptors, respectively. Released SST can bind to SSTR subtypes 1–5 expressed on postsynaptic neurons and then inhibit calcium influx. This eventually leads to the reduced excitability of postsynaptic neurons through a downstream signaling pathway.

It has been reported that neurons can release SST in a calcium-dependent manner^30^, even in the absence of exogenous stimuli such as sensory information^31^. Other studies have shown that membrane depolarization^32^, glutamate^33^, or NMDA application^34^ can stimulate SST release via activation of NMDA and AMPA receptors (Fig. 3). Along with in vitro experiments, several attempts to dissect the mechanism of SST release in vivo have led to the identification of factors that can modulate SST release. Striatal SST^+^ INs can corelease glutamate and GABA, generating excitation–inhibition sequences in postsynaptic neurons, as the glutamatergic response persists for a shorter period than the inhibitory response^35^. The corelease of GABA and glutamate from striatal SST^+^ INs is evoked by glutamate-induced activation of ionotropic AMPA/NMDA receptors that are expressed in axon terminals. Cotransmission of these two neurotransmitters is induced in the striatum but not in the cortex or the hippocampus. Cortical SST^+^ INs are known to corelease GABA and SST onto postsynaptic neurons upon activation^36,37^. Interestingly, GABA release from SST^+^ INs inhibits the release of GABA as well as the spontaneous release of SST^31^. This autoregulation is induced by GABA_B_ receptors, which are expressed on the axon terminals of SST^+^ INs^38^ (Fig. 3). SST^+^ INs also express SSTR1 in the cortex (Fig. 2), and binding of SST to SSTR1 can modulate the further release of SST or GABA from SST^+^ INs. Future studies are required to understand the exact molecular mechanisms of the corelease of GABA and SST and how SST release modulates the excitability of postsynaptic neurons in vivo.

## Decrease in SST expression in various neurological disorders

**Fig. 4:**
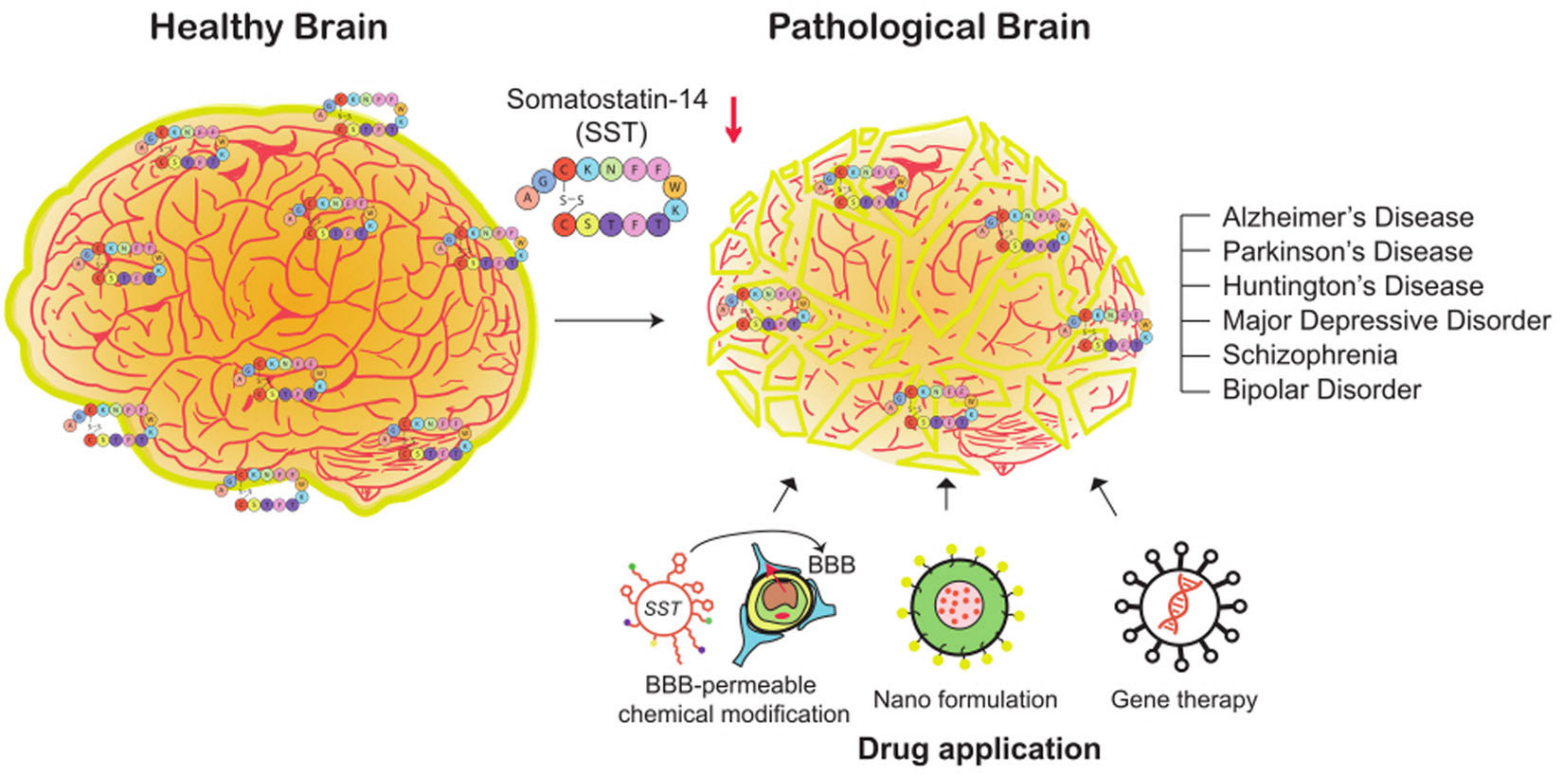
Decrease in SST expression in the brain in the context of various neurological disorders. In the normal human brain (left), SST is highly expressed throughout the brain and in the cerebrospinal fluid. The most abundant SST isoform in the brain is SST-14, which contains 14 amino acids with a disulfide bond between cysteine residues. In the context of various neurological disorders (right), alterations in SST expression in a specific region or throughout the brain are observed. A decrease in SST expression might lead to a shrinkage of the brain and an imbalance in neural networks and function. The following disorders showing such pathologies are discussed in the text: Alzheimer’s disease, Parkinson’s disease, Huntington’s disease, major depressive disorder, and schizophrenia. Three approaches can be used to deliver functional neuropeptide SST to the brain for the treatment of these disorders: chemical modification to enhance BBB penetration, nanoformulations, and gene therapy techniques.

It has been reported that neurodegenerative and neuropsychiatric disorders such as Alzheimer’s disease (AD), Parkinson’s disease (PD), Huntington’s disease (HD), major depressive disorder (MDD), bipolar disorder, and schizophrenia (SCZ) are linked to a decrease in the expression of SST (Fig. 4). Here, we summarize these findings and describe the relationship between SST expression in the brain and neurological disorders.

### Alzheimer’s disease

It has been repeatedly reported that SST expression is reduced in AD patients and mouse models, both in the brain and the cerebrospinal fluid (CSF)^39^. In AD patients, total SST expression levels and the number of neurons expressing SST in the frontal cortex and hippocampus were decreased to less than 30% of those in control subjects^39^. In the hippocampus, the loss of SST^+^ neurons and the reduction in SST mRNA expression occurred earlier than the alteration in the expression of other GABAergic, glutamatergic, and cholinergic markers or the number of principal neurons^40^. Although the number of cortical and hippocampal SST^+^ INs was markedly reduced in AD^41^, the changes in the expression of different SSTR subtypes were not consistent^42^. In the AD cortex, the expression of SSTR4 and SSTR5 was significantly reduced, SSTR2 immunoreactivity showed a modest decrease, and SSTR1 seemed unaffected. Conversely, the expression of SSTR3 was increased in the frontal cortices of AD patients^42^. Therefore, the reduction in SST expression in the AD brain is more consistent than that in SSTR expression. It has been shown that an increase in the level of Aβ peptide is linearly correlated with SST deficiency^43^, indicating that a decrease in the expression of SST is likely involved in amyloid accumulation in the context of AD pathogenesis. We will further discuss this phenomenon below (“Disruption in SST function causes AD pathogenesis”).

### Parkinson’s disease

PD is caused by the loss of dopaminergic neurons in the substantia nigra pars compacta and the locus coeruleus, leading to dopamine depletion that causes the gradual onset and progression of motor and nonmotor symptoms^44^. The molecular pathological mechanisms that underlie PD are the accumulation of α-synuclein (αSyn) and the formation of Lewy bodies^45^. In PD, accumulation of αSyn occurs throughout the brain, including both cortical and subcortical areas as well as the central and peripheral (autonomic) nervous systems^46^. Interestingly, changes in SST expression and SSTRs in cortical regions (the frontal and entorhinal cortices and hippocampus) and the CSF have been demonstrated in PD^47^. A decrease in SST levels in these cortical areas can be the putative cause of cognitive impairments. One recent paper showed a decrease in the number of SST^+^ INs and SST mRNA levels in PARK2-specific iPSCs derived from PD patients^48^. A PARK2 mutation in SST^+^ INs may cause a decrease in SST transcripts and mitochondrial dysfunction, which might cause an excitatory/inhibitory (E/I) imbalance culminating in the motor and nonmotor symptoms observed in PD^49^.

### Huntington’s disease

HD is a genetic disorder that is caused by the expansion of CAG trinucleotide repeats in exon 1 of the *huntingtin (HTT)* gene on chromosome 4 over a certain threshold (>39 repeats). The translation of this mutated gene results in the production of mutant HTT protein (mHTT), which has toxic effects and causes pathological changes in neurons, such as synaptic dysfunction and axonal transport impairment^50^. HD’s characteristic neuropathological feature is atrophy of the striatum, cerebral cortex, hippocampus, thalamus, hypothalamus, and cerebellum^51^. Massive degeneration and loss of spiny projection neurons in the striatum are observed, which might disrupt the relay of information from the cortex and the thalamus to the output structures of the basal ganglia^52^. A reduced number of PV^+^, SST^+^, and cholinergic INs in addition to spiny neurons, a reduction in dendritic arborization, and altered physiology are observed in HD mice^53,54^. In particular, SSTR1 and SSTR5 double knockout mice were found to exhibit neurochemical changes that mimic those observed in HD^55^. In addition, postmortem analysis of HD patients showed a reduction in the number of SST^+^ neurons in the nucleus tuberalis lateralis of the hypothalamus^56^.

### Major depressive disorder

MDD is accompanied by persistent changes in various cognitive functions, such as attention, short-term and working memory^57^, and cognitive control^58^. In MDD, SST levels are decreased in the CSF, and the level of SST expression is restored to the normal level when patients recover from MDD^59^. Low levels of SST expression in the CSF were correlated with elevated levels of urinary cortisol in MDD patients, who also exhibited hypothalamic–pituitary–adrenal (HPA) dysfunction^60^. However, it is unclear whether a decrease in SST expression causes MDD pathophysiology. A tendency for SST expression to be downregulated in the CSF and brain areas such as the ACC^61^ and amygdala^62^ was observed in human postmortem studies. Interestingly, females showed higher vulnerability to MDD development and a greater reduction in SST expression in the cortex and amygdala than males^63,64^. Future studies are required to understand the molecular function of SST in MDD pathology.

### Schizophrenia

SCZ is a neuropsychiatric disorder characterized by positive (e.g., hallucinations and delusions), negative (e.g., blunted affect, apathy, and social avoidance), and cognitive (e.g., deficits in attention and executive function) symptoms. The most common cause of positive symptoms in SCZ is excessive subcortical dopamine release, considering that D2 receptor antagonists reduce positive symptoms and thus are used as antipsychotics^65^. Although no observable primary pathology has been identified in the dopamine system in SCZ patients, it has been postulated that upstream areas of the dopamine system are impaired in SCZ, such as the ventral hippocampus^66^. Indeed, hyperactivity of the ventral hippocampus has been observed in SCZ patients, and it has been suggested that this might be the result of a loss of INs, such as PV^+^ and SST^+^ INs^67^. Reduced expression of SST in SCZ patients was observed not only in the CSF^68^ but also in the hippocampus, thalamic reticular nucleus, and cortical areas^67,69^. Additionally, in a postmortem study of SCZ patients, neurochemical changes accompanied a reduction in SST levels in the lateral amygdala^69^. As shown in an SCZ mouse model with a mutation in the region corresponding to human chromosome 16p11.2 (16p11.2 duplication mice)^70^, disruption of hippocampal–orbitofrontal and hippocampal–amygdala functional connectivity in the SCZ correlates with a reduction in SST expression.

## Disruption of SST function in AD pathogenesis

Considering that AD patients exhibit low SST expression in the cortex and hippocampus^39^, a causal link between SST function and AD pathogenesis has been postulated. The main symptom of AD is gradual but severe memory loss. Numerous studies have reported that memory loss in AD patients may have been derived from deficits in SST function. Electroconvulsive shock-induced amnesia in rodents performing an active avoidance task was reversed after intracerebroventricular injections of SST^71^. In AD patients, SST infusion into the brain and systemic SST administration improved cognitive defects. Craft et al.^72^ further showed that catheter-mediated intravenous (IV) administration of octreotide, which is an analog of SST and is known to activate SSTR2, SSTR3, and SSTR5, improved memory loss.

Interestingly, SST enhanced the enzyme activity of neprilysin, which promotes Aβ degradation and is downregulated with aging and in the early stage of AD progression^73^. In a study using amyloid precursor protein (APP) transgenic mice^74^, a well-known AD mouse model, amyloid plaque formation and embryonic lethality in mutant mice were fully reversed by overexpression of neprilysin. Furthermore, delivery of neprilysin to the presynaptic site using a recombinant adeno-associated viral vector blocked Aβ deposition in the hippocampi of APP-transgenic mice and neprilysin-deficient mice^75^. SST enhanced neprilysin expression in cultured murine neurons but decreased Aβ42 expression via binding to its receptors^76^. Similarly, blocking the function of SSTR by administering BIM23056 (an SSTR5 antagonist) or pertussis toxin (a GPCR blocker that inhibits adenylyl cyclases) reduced these effects. Moreover, in SST knockout (KO) mice, there was a 50% decrease in neprilysin activity and an increase in Aβ42 accumulation^76^. These findings demonstrate the correlation between the expression levels of SST, neprilysin, and Aβ42, which are important pathological hallmarks for diagnosing AD. However, in some of the AD mouse models, such as APPswe/PS1dE9 mice, the SST level was increased or remained constant despite the disruption in cognitive brain function and the occurrence of amyloid deposition^77^. Future studies are required to identify the direct causal relationships between SST and AD pathology.

Hyperphosphorylation of tau proteins is another key factor in AD pathogenesis. Tau proteins regulate the assembly and organization of microtubules. Phosphorylation of the tau protein weakens its affinity for microtubules and subsequently induces the depolymerization of microtubules^78^. Furthermore, there is increasing evidence that an increase in Aβ fibrils results in tau phosphorylation in cultured hippocampal and cortical neurons^79^. In AD pathogenesis, the phosphorylation, polymerization, and deposition of the tau protein are facilitated in affected brain areas^80^. Interestingly, SST treatment decreased the phosphorylation of tau at Ser262, a site that is known to be affected in the AD brain^81^. The phosphorylation of the tau protein at Ser262 via activation of SSTR2 and SSTR4 was observed in the cortex not in other brain areas.

## SST analogs developed for therapeutic applications

SST exerts potent inhibitory effects on a wide range of endocrine and exocrine systems in the body. It is well known to inhibit the secretion of growth hormone to suppress body growth. Nevertheless, its ability to be readily degraded in its natural form, along with its short half-life (1–3 min), hinder its application as a drug for various diseases. Several SST analogs (SSAs) that show enhanced stability in the body have been developed. They share a similar structure and function with SST but show distinct receptor-binding properties (Fig. 1). One well-known natural analog of SST is cortistatin (CST), which has been identified as a natural neuropeptide expressed in the cortex that bears the same amino acid sequence in the receptor-binding site as SST. CST can bind to all subtypes of SSTRs with nanomolar affinity and has a functionally similar role as SST in that it also suppresses neural activity^82^. Due to the difference in tissue expression patterns and the detailed molecular structures, CST and SST exhibit different functions. For example, CST, but not SST, can enhance slow-wave activity^82^ and is able to consolidate short- and long-term memories^83^. These differences may be due to differences in molecular partners activated by CST and SST. Despite the different roles of CST, cortical injection of CST was shown to induce the same enhancement of perceptual behaviors in mice as SST^27^. Future studies are required to compare and understand the roles of SST and CST in the cortex in vivo.

In addition to CST, five synthetic SSAs have been further developed for the treatment of disorders: SMS 201–995 (octreotide), BIM 23014 (lanreotide), RC-160 (vapreotide), MK 678 (seglitide), and SOM 230 (pasireotide). Three of these SSAs (octreotide, lanreotide, and pasireotide) were approved by the U.S. Food and Drug Administration (FDA) and the European Medicines Agency (EMA) for the clinical treatment of disorders characterized by excessive release of growth hormone. Octreotide acetate and lanreotide are first-generation SSAs, and have a longer half-life (2–600 h) than SST^84,85^. Octreotide is an octapeptide that evokes similar effects as SST but exerts more variable, prolonged, and selective inhibitory effects on target tissues. Octreotide and lanreotide specifically show strong binding affinity for SSTR2 and SSTR5. On the other hand, pasireotide is a second-generation SSA that is also known as Signifor. The first version of this orphan drug, Signifor® (Novartis, Geneva), has a 9.6- to 12.6-h half-life. The improved version, Signifor® LAR (Novartis, Geneva), has a much longer half-life (375–443 h)^86^. This analog has a higher binding affinity for SSTR1, SSTR3, and SSTR5 than octreotide but a similar affinity for SSTR2 (ref. ^86^) (Fig. 1).

Some SSAs have a receptor-activating motif (Phe–Trp–Lys–Thr) as well as another motif that can strongly suppress hormonal secretion. These modifications are useful for treating hyperhormonal diseases, including neuroendocrine tumors and acromegaly^87,88^. For example, pasireotide is used to treat acromegaly patients who do not respond to first-generation SSAs^89^. SSAs are also applied to treat various diseases, such as pituitary adenomas and gastroenteropancreatic neuroendocrine tumors. For example, SSAs, as inhibitors of adrenocorticotropic hormone secretion, can be used to treat Cushing’s disease, a form of pituitary adenoma^89^.

Recently, the development of SSA drugs has focused on chemical formulations for oral delivery. The SSAs approved thus far must be administered by injected subcutaneous or intramuscular injection, which are accompanied by discomfort and pain, leading some patients to delay or skip treatments. Two important issues that must be taken into account when designing oral formulations of SSAs are their stability in the presence of gastrointestinal (GI) peptidases and their ability to pass through the intestine to reach the blood vessels. Since most existing SSAs are stable in the presence of GI enzymes, the key issue is their poor ability to pass through the epithelium (<0.3%) of the small intestine^90^. As several attempts to enhance the ability of orally administered drugs to pass through the intestine have been made^91^, future application of these techniques may be useful for developing SSA drugs for oral delivery.

## Development of SST-related drugs for the treatment of brain disorders in the future

Although SSAs have been used for hormone regulation in the body, there are still no SSA drugs that are approved for the treatment of brain disorders. The first hurdle for the application of SSAs for the treatment of brain disorders is the poor ability of SSAs to enter the brain. The use of iodinated Tyr-SST administered via carotid artery injection^92^ and octapeptide analogs of SST administered via IV injection^93^ is hindered by the limited ability of these drugs to penetrate the brain. Indeed, the greatest challenge to the development of drugs for neurological disorders is the difficulty in delivering drugs across the blood–brain barrier (BBB) and the blood–CSF barrier to the brain^94^. Although the BBB is a tight barrier that prevents large substances from entering the brain, some viruses can easily penetrate the BBB by the biological mechanisms of receptor binding and tranport^95^. Thus, many researchers have used such mechanisms to develop drug delivery systems. The chemical motifs of drugs are modified to allow them to bind the surface receptors of endothelial cells and be transported into the brain^96^ (Fig. 4).

For the delivery of therapeutic drugs across the BBB, nanotechnology-based engineering approaches that introduce desired functions to packaged drugs using the unique physicochemical properties of biocompatible and biodegradable nanomaterials have also been considered^97^. Nanocarriers such as micelles, liposomes, polymeric nanoparticles, solid lipid nanoparticles, nanoemulsions, and dendrimers are currently being developed^94^. Several studies have investigated the systemic delivery of neuroprotective peptides loaded in nanoparticles and showed improved brain functions in rodent models^98^. Similarly, encapsulation of SSAs may be useful for developing SSA drugs that can improve brain functions. Since many neurological disorders linked to SST function are associated with a reduction in SST expression, direct delivery of SSAs as well as gene therapy techniques can be used to express genetically encoded SSAs in the brain more permanently (Fig. 4). Future studies are required to design gene delivery methods that can target specific neuronal and nonneuronal cells for long-term expression of SSAs in the brain.

## Concluding remarks

In this paper, we summarized the pivotal functions of SST and its receptors in the brain, especially in cortical processing. We also described their relationships with various neurological disorders, including AD. We further discussed and suggested possible strategies for developing SSAs that can be used to treat neurological disorders. Regardless of the numerous attempts that have been made to deliver peptide drugs to the CNS, several barriers, such as chemical stability, receptor sensitivity, and BBB permeability, remain major challenges to developing more efficient SSA drugs. In future studies, chemical modification, encapsulation in biocompatible and biodegradable materials or nanovesicles, and delivery of SSA genes may be used to overcome these barriers and stably deliver SST-related drugs to the brain.
